# Association of polymorphisms of *PTEN*, *AKT1*, *PI3K*, *AR*, and *AMACR* genes in patients with prostate cancer

**DOI:** 10.1590/1678-4685-GMB-2018-0329

**Published:** 2020-06-01

**Authors:** Monyse de Nóbrega, Heloisa Lizotti Cilião, Marilesia Ferreira de Souza, Milene Roldão de Souza, Juliana Mara Serpeloni, Paulo Emilio Fuganti, Ilce Mara de Syllos Cólus

**Affiliations:** 1 Universidade Estadual de Londrina (UEL), Departamento de Biologia Geral, Londrina, PR, Brazil; 2 Hospital do Câncer de Londrina, Londrina, PR, Brazil

**Keywords:** SNP, prognosis, rs2494750, rs2735343, rs3195676

## Abstract

Polymorphic variants in the *PTEN* (rs2735343), *PI3K* (rs2699887), *AKT1* (rs2494750), *AR* (rs17302090), and *AMACR* (rs3195676) genes were evaluated as possible molecular markers of susceptibility, prognosis, and progression of prostate cancer (PCa), in a case-control study. Samples consisted of 277 patients with PCa and 277 controls from Londrina, PR, Brazil. SNPs were analyzed by real-time PCR. A family history of cancer, including PCa, as well as level of schooling were risk factors for PCa. The data were obtained via logistic regression, using odds ratios with a CI 95%. The genotypes of *AKT1* and *AKT1+AR* demonstrated an association with protection for the disease. The combination of SNPs with the histopathological tumor data between allele variants of *AMACR, AKT1+AR*, and *AKT1+AMACR* indicated an association with protection against seminal vesicle invasion. The polymorphisms *AKT1+AR and PI3K+AR* were associated with protection against tumor bilaterality. The genotype combinations *PTEN+AMACR* and *PTEN+AR* were associated with the risk of extracapsular extension. Of the five genes studied, two were associated with protection for PCa, four were associated with protection for some prognostic variables, and only one was associated with risk. Thus, these SNPs are candidates for markers to discriminate men with better or worse prognosis for PCa.

## Introduction

Prostate cancer (PCa) is a heterogeneous and multifactorial pathology that presents itself in the form of indolent or very aggressive tumors and is a significant source of morbidity in the male population. In 2018, 1.3 million new cases were estimated and 359,000 associated deaths worldwide, however, the mortality rates do not follow the incidence rates. In certain countries, PCa still presents an elevated degree of mortality (Saini, 2016; Bray *et al.*, 2018).

The most commonly utilized methods for the prognosis of this type of tumor are the TNM classification for staging, used in combination with the Gleason/tumor grade and prostate-specific antigen (PSA) level (Buyyounouski *et al.*, 2017). However, the clinical applications of these indicators are limited. The American Urological Association encourages the identification of novel markers that can identify men at a greater risk of developing and progressing in the disease (Carter *et al.*, 2013). Studies have increasingly associated single-nucleotide polymorphisms (SNPs) with various diseases, resulting in SNPs being progressively utilized as markers associated with cancer, including PCa. Consequently, SNPs have received considerable attention for use as prognostic indicators in pre and post-treatment PCa patients (Qi *et al.*, 2017).

In recent decades, different SNPs have been associated with important cellular pathways, including the PI3K/PTEN/AKT signaling pathway (Jang *et al.*, 2013), androgen action pathway (Prencipe *et al.*, 2015), and fatty acid metabolization pathway (Jiang *et al.*, 2013). In this manner, they are related to the development of prostate adenocarcinoma.

The *PTEN* gene is a tumor suppressor with dual specificity. The principal substrate is phosphatidylinositol (3,4,5)-trisphosphate (PIP3). The protein PTEN acts as an antagonist on the PI3K/AKT pathway by maintaining low PIP3 levels. The greater the concentration of PIP3, the greater the signaling of the PI3K/AKT pathway, which promotes inhibition of the cellular cycle, migration, metastasis, and apoptosis. The occurrence of loss of *PTEN* gene function is greater in aggressive metastatic disease, meaning that it can be used as a biomarker for prognosis in distinguishing indolent tumors from those that are aggressive (Di Cristofano and Pandolfi, 2000; Jang *et al.*, 2013; Wise *et al.*, 2017).

Meanwhile, the *PI3K* gene is a proto-oncogene; the corresponding protein phosphorylates phosphatidylinositol 4,5-bisphosphate (PIP2), converting it into PIP3. Given the complexity of this intracellular signaling cascade and its association with the development of PCa, it is important to delineate the role of polymorphism in this gene with respect to prognosis and susceptibility (Toren and Zoubeidi, 2014).

In addition, the *AKT1* gene is a proto-oncogene whose corresponding protein regulates events downstream in the PI3K signal transduction pathway. This protein plays an important role in inhibition of cellular viability, proliferation, differentiation, and apoptosis (Vivanco and Sawyers, 2002). The overexpression of this gene is related to different types of cancer, including the progression and biochemical recurrence of prostate tumors (Feldman and Feldman, 2001; Karyadi *et al.*, 2015).

Finally, the *AR* gene encodes the androgen receptor, a nuclear transcription factor that is involved in the development, maintenance, and regulation of the male reproductive system (Feldman and Feldman, 2001). Mutations in *AR* are associated with a series of diseases, ranging from androgen insensitivity syndrome to prostate cancer (Heemers and Tindall, 2007). Among the mutations in this gene, SNPs are the most frequent type of alteration. Even a simple change in amino acids can result in either an increase in expression or a loss in gene function (Sun *et al.*, 2010; Wright *et al.*, 2011).

The peroxisomal and mitochondrial enzyme Alpha-methylacyl-CoA racemase (AMACR) is coded by the gene *P504S* (*AMACR*) and is involved in the biosynthesis and beta-oxidation of dietary fatty acids (Li *et al.*, 2014). Fatty acid metabolism represents a key process that influences different pathways and cellular characteristics, including cellular signaling, energetic processing, and membrane fluidity, among others (Liu *et al.*, 2006).

Considering that the majority of cases of PCa are indolent, markers are needed that aid in classifying the degree of tumor aggressiveness and in providing individual treatment, in line with the genetic characteristics of each patient. In this sense, a case-control study was performed that evaluated the possible relationship between polymorphic variants of the genes *PTEN, PI3K, AKT1, P504S (AMACR)*, and *AR* and prostate cancer susceptibility. In addition, data from the association study were correlated with the clinical parameters of the patients and the histopathological parameters of the tumors, with the aim of establishing prognostic molecular markers.

## Subjects and Methods

### Study cohorts

In total, 554 male individuals were studied, with 277 serving as a control, free of PCa with a prostate-specific antigen (PSA) value lower than 2 ng/mL, and with 277 patients diagnosed with PCa, as confirmed by histopathological analysis. Individuals were excluded if they had been submitted to radiotherapy and/or chemotherapy. The patients were paired with controls on a 1:1 basis, maintaining age (± 5 years), ancestry (European and African), and alcohol and tobacco use consistent between pairs. Peripheral blood samples of the patients and controls were collected from 2006 to 2015 at CISMEPAR (Consórcio Intermunicipal de Saúde do Médio Paranapanema), the Irmandade Santa Casa de Londrina, the State University of Londrina, and the Cancer Hospital of Londrina.

Disease stage was determined by pathology findings, pelvic computed tomography, or magnetic resonance image. Tumor staging was determined according to criteria established by the Union for International Cancer Control (2009) tumor-node-metastasis (TNM) classification system. Pathologic grading was recorded as the Gleason score and was further classified into 2 groups: Gleason score 3–7 (3+4) and Gleason 7(4+3)–10.

The patients and controls were volunteers and written informed consent was obtained from each participant that responded to the questionnaire, which was based on Carrano and Natarajam, (1988). The study was approved by the Ethics Committee Involving Research on Human Beings of the State University of Londrina (CEPE/UEL 176/2013).

### Extraction of genomic DNA

Peripheral blood was collected in tubes of EDTA (ethylenediaminetetraacetic acid), from which DNA was extracted using the High Pure PCR Template Preparation (Cat. 11796828001; Roche; IN, USA) commercial kit, following the manufacturer's instructions. DNA quantification was carried out via spectrophotometry, using a Nanodrop 2000 UV-Vis Spectrophotometer (Thermo Fisher Scientific, Wilmington, DE, USA). Absorption was measured at 260/280 nm in order to determine the purity of the nucleic acid, with samples with a ratio between 1.8 and 2.0 being considered pure.

### Genotyping

Each gene selected for this study had one defined SNP chosen from the public Database of Single Nucleotide Polymorphisms (NCBI, 2016): *PTEN* (rs2735343), *AKT* (rs2494750), *PI3K* (rs2699887), *AR* (rs17302090), and *P504S (AMACR)* (rs3195676).

The genotypes were determined via real-time PCR molecular analysis using a hydrolysis probe (TaqMan System, Applied Biosystems, Foster City, CA, USA). The reactions contained the genotyping master mix (Applied Biosystems), 5 ng/mL of DNA, and the specific probe for each gene. Reagent quantities and cycling were carried out according to the manufacturer's specifications.

### Statistical analysis

The χ^2^ test was used to determine if the genotype distributions were in a Hardy-Weinberg equilibrium. Allele frequency was calculated as [1(h + 2H)]/2 N, where h represents the heterozygous genotype, H is the homozygous genotype, and N is the sample size for each population.

First, the association between the following parameters: family history of cancer, family history of PCa, vasectomy, occupational exposure to agrochemicals, level of schooling, and consumption of red meat was determined through univariate logistic regression analysis, aiming to assess the impact of these variables on cancer risk. The association between the genotypes and cancer risk was also run by univariate logistic regression analysis.

Subsequently, multivariate logistic regression analyses were performed aiming to assess the impact of the studied SNPs on PCa risk. Variables that had a *p*-value ≤ 0.05 were included in the model: family history of cancer, family history of PCa, and level of schooling, avoiding possible bias in the analysis.

The different genotypes were evaluated with respect to histopathological parameters (Gleason score, bilaterality, seminal vesicle invasion, perineural invasion, lymphatic invasion, and extracapsular extension) and a clinical parameter (PSA).

Results are expressed as Odds Ratio (OR) with a confidence interval of 95% (CI95%) and assumed statistically significant for a *p* ≤ 0.05. Analyses were performed using SPSS 20.0 (IBM, Armonk, NY, USA).

## Results

The total sample was composed of 554 men, of which 85.6% were of European and 14.4% of African descent. The average age of the patients was 65.38 ± 7.18 years, while that of the control was 63.65 ± 8.21 years. In both groups, 68 individuals (24.5%) were tobacco users, while 141 individuals (50.9%) consumed alcohol.

Genotype frequencies were in Hardy-Weinberg equilibrium within a significance level of 5%. The univariate statistical analysis revealed significant differences between patients and controls with respect to a family history of cancer (58.8% of patients had at least one first-degree relative who was affected), a family history of PCa, and level of schooling. Other analyzed parameters, such as vasectomy, occupational exposure to agrochemicals, and red meat consumption, did not present statistically significant differences between patients and controls (Table 1).

**Table 1:** Demographic and clinical characteristics of prostate cancer patients and cancer-free controls.

Characteristics		Case		Controls		*P*
		N	(%)	N	(%)	
Family history of cancer	No	114	(41.2)	159	(57.4)	
	Yes	163	(58.8)	118	(42.6)	**0.000** *
	Total	277	(100.0)	277	(100.0)	
Family history of PCa	No	101	(65.2)	86	(77.5)	
	Yes	54	(34.8)	25	(22.5)	**0.031** *
	Total^a^	155	(100.0)	111	(100.0)	
Vasectomy	No	270	(98.5)	265	(97.4)	
	Yes	4	(1.5)	7	(2.6)	0.361
	Total^a^	274	(100.0)	272	(100.0)	
Occupational exposure to agrochemicals	No	115	(41.5)	134	(48.4)	
	Yes	162	(58.5)	143	(51.6)	0.105
	Total^a^	277	(100.0)	277	(100.0)	
Level of schooling	S	47	(17.0)	45	(16.2)	
	P	167	(60.3)	20	(72.6)	0.327
	N	61	(22.0)	27	(9.7)	**0.013** *
	Total^a^	275	(100.0)	92	(100.0)	
Consumption of red meat	No	9	(3.2)	8	(2.9)	
	Yes (1-4)	151	(54.5)	137	(49.5)	0.967
	Yes (5-7)	117	(42.2)	132	(47.7)	0.635
	Total	277	(100.0)	277	(100.0)	

a It was not possible to evaluate all patients and controls. Level of schooling - S: secondary school complete or higher; P: primary school incomplete to secondary school incomplete; N: no formal schooling. Consumption of red meat - 1-4: 1 to 4 times per week; 5-7: 5 to 7 times per week.

* significant *p*-values are in bold.

The comparison of the genotypic variants and allele frequencies of each of the genes studied showed no statistically significant differences between patients with PCa and controls. A multivariate statistical analysis adjusted for the determined risk factors indicated a protecting effect for genotypes CG and CG + GG for the rs2494750 *AKT1*gene (Table 2). A multivariate analysis of the paired genes (Table S1) showed that the genotypes *AKT1* (CG) + *AR* (A) provided protection against PCa (Table 3).

**Table 2:** Univariate and multivariate regression analysis of genotypes and allele of *PTEN, AKT1, PI3K, AR*, and *AMACR* genes in controls and patients with prostate cancer.

Gene	Genotypes	Case N (%)	Control N (%)	Not adjusted		Adjusted	
SNP_ID				OR (CI95%)	*p*	OR (CI95%)	*p*
*PTEN*	CC	55 (19.9)	36 (13.0)	Reference		Reference	
rs2735343	CG	115 (41.5)	143 (51.6)	**0.53 (0.32-0.86)**	**0.010** *	0.44 (0.17-1.15)	0.092
	GG	107 (38.6)	98 (35.4)	0.72 (0.43-1.18)	0.189	0.58 (0.22-1.54)	0.275
Dominant model							
	CC	55 (19.9)	36 (13.0)	Reference		Reference	
	CG+GG	222 (80.1)	241 (87.0)	**0.60 (0.38-0.95)**	**0.030** *	0.50 (0.21-1.20)	0.121
Recessive model							
	CC+CG	170 (61.4)	179 (64.6)	Reference		Reference	
	GG	107 (38.6)	98 (35.4)	1.15 (0.81-1.62)	0.428	1.08 (0.59-1.98)	0.800
Allele frequency							
	C	225 (40.6)	215 (38.8)	Reference			
	G	329 (59.4)	339 (61.2)	0.93 (0.73-1.18)	0.539		
							
*PI3K*	GG	172 (62.1)	177 (63.9)	Reference		Reference	
rs2699887	GA	90 (32.5)	87 (31.4)	1.07 (0.74-1.53)	0.735	0.95 (0.51-1.75)	0.859
	AA	15 (5.4)	13 (4.7)	1.18 (0.56-2.48)	0.663	1.50 (0.36-6.24)	0.579
Dominant model							
	GG	172 (62.1)	177 (63.9)	Reference		Reference	
	GA+AA	105 (37.9)	100 (36.1)	1.08 (0.77-1.53)	0.660	1.00 (0.55-1.81)	0.999
Recessive model							
	GA+GG	262 (94.6)	264 (95.3)	Reference		Reference	
	AA	15 (5.4)	13 (4.7)	1.16 (0.54-2.49)	0.698	1.56 (0.81-3.02)	0.188
Allele frequency							
	G	433 (78.2)	441 (79.6)	Reference			
	A	121 (21.8)	113 (20.4)	1.09 (0.82-1.46)	0.556		
*AKT1*	CC	210 (75.8)	203 (73.2)	Reference		Reference	
rs2494750	CG	66 (23.8)	73 (26.4)	0.87 (0.60-1.28)	0.493	**0.75 (0.57-0.99)**	**0.045** *
	GG	1 (0.4)	1 (0.4)	0.97 (0.06-15.56)	0.981	–	–
Dominant model							
	CC	210 (75.8)	203 (73.2)	Reference		Reference	
	CG+GG	67 (24.2)	74 (26.7)	0.88 (0.60-1.28)	0.495	**0.76 (0.58-0.99)**	**0.044** *
Recessive model							
	CG+CC	276 (99.6)	276 (99.6)	Reference		Reference	
	GG	1 (0.4)	1 (0.4)	1.00 (0.06-16.07)	1.000	–	–
Allele frequency							
	C	486 (87.7)	479 (86.5)	Reference			
	G	68 (12.3)	75 (13.5)	0.89 (0.63-1.27)	0.531		
							
*AMACR*	GG	107 (38.7)	96 (34.7)	Reference		Reference	
rs3195676	GA	125 (45.1)	118 (42.6)	0.95 (0.65-1.38)	0.789	1.02 (0.54-1.94)	0.948
	AA	45 (16.2)	63 (22.7)	0.64 (0.40-1.03)	0.064	0.53 (0.24-1.19)	0.124
Dominant model							
	GG	107 (38.6)	96 (34.7)	Reference		Reference	
	GA+AA	170 (61.4)	181 (65.3)	0.84 (0.60-1.19)	0.332	0.84 (0.45-1.56)	0.575
Recessive model							
	GA+GG	232 (83.8)	214 (77.3)	Reference		Reference	
	AA	45 (16.2)	63 (22.7)	0.66 (0.43-1.01)	0.054	0.54 (0.27-1.04)	0.067
Allele frequency							
	G	339 (61.2)	310 (56.0)	Reference			
	A	215 (38.8)	244 (44.0)	0.81 (0.63-1.02)	0.077		
							
*AR*	G	99 (35.7)	88 (31.8)	Reference		Reference	
rs17302090	A	178 (64.3)	189 (68.2)	0.84 (0.59-1.19)	0.323	0.81 (0.49-1.35)	0.422

OR (CI95%): odds ratio value, with a confidence interval of 95%. Adjusted for family history of cancer, family history of prostate cancer, and schooling.

* Statistically significant value, *p*< 0.05; in bold.

**Table 3:** Univariate and multivariate logistic regression analysis of the association between *AKT1* and *AR* genes in prostate cancer patients and controls.

Gene	Genotypes	Cases N (%)	Controls N (%)	Not adjusted		Adjusted	
SNP_ID				OR (CI95%)	*p*	OR (CI95%)	*p*
*AKT1*	CC+G	71 (25.6)	66 (23.8)	Reference		Reference	
rs2494750	CC+A	139 (50.2)	137 (49.5)	0.94(0.63-1.42)	0.780	1.01 (0.62-1.64)	0.965
**+**	CG+G	28 (10.1)	22 (7.9)	1.83 (0.62-2.27)	0.613	1.28 (0.62-2.66)	0.505
*AR*	CG+A	38 (13.7)	51 (18.4)	0.69 (0.41-1.19)	0.180	**0.50 (0.26-0.97)**	**0.041** *
rs17302090	GG+G	0 (0.0)	0 (0.0)	0.93 (0.06-15.17)	0.959	–	–
	GG+A	1 (0.4)	1 (0.4)	–	–	–	–

OR (CI95%): odds ratio value, with a confidence interval of 95%. Adjusted for family history of cancer, family history of prostate cancer, and schooling.

* Statistically significant value, *p*< 0.05.

Table 4 presents the clinical and histopathological parameters obtained from the patient histories. In total, 147 patients presented a PSA level of 4.1-10.0 ng/mL, 115 patients presented a level greater than 10.0 ng/mL, and only 10 had a level below 4.0 ng/mL. Meanwhile, 133 patients had Gleason scores of 3-6, 117 patients had a score of 7, and only 25 had scores of 8-10. Tumor bilaterality was observed in 137 patients. According to the TNM system, 154 cases had tumors in stage T2, while 101were in stages T3 and T4. The other parameters were absent for the majority of the patients.

**Table 4:** Analysis of PSA levels of prostate cancer patients and histopathological characteristics of the tumors.

Parameters	Categories	N	%
PSA level	0.0 - 4.0	10	3.7
(ng/mL)	4.1 - 10.0	147	54.0
	> 10.1	115	42.3
	Total*	272	100.0
Gleason Score	3-6	133	48.4
	7	117	42.5
	8-10	25	9.1
	Total*	275	100.0
Extracapsular extension	Presence	97	37.7
	Absence	160	62.3
	Total*	257	100.0
Seminal vesicle invasion	Presence	32	12.5
	Absence	225	87.5
	Total*	257	100.0
Perineural invasion	Presence	42	16.3
	Absence	215	83.7
	Total*	257	100.0
Bilaterality	Presence	137	53.1
	Absence	121	46.9
	Total*	258	100.0
Lymphatic invasion	Presence	6	2.5
	Absence	232	97.5
	Total*	238	100.0
TNM Staging	T2	154	60.4
	T3 and T4	101	39.6
	Total*	255	100.0

* It was not possible to evaluate the parameters for all 277 patients.

The genotypes of the five SNPs were evaluated for their relationship with the various histopathological and clinical parameters (Table S2). A comparison of the heterozygous and/or polymorphic genotypes with the prevalent genotypes indicated that only the isolated GA and GA+AA genotypes of the *AMACR* gene protected against seminal vesicle invasion (Table 5).

**Table 5:** Univariate regression analysis between genotypes of *PTEN, AKT, PI3K, AR*, and *AMACR* genes with clinicopathological features in prostate cancer patients.

Histopathological parameters	Genes SNP_ID	Genotypes	Present N (%)	Absent N (%)	OR (CI95%)	*p*
Seminal vesicle	*AMACR*	GG	19 (59.4)	82 (36.4)	Reference	
Invasion	rs3591676	GA	8 (25.0)	105 (46.7)	**0.33 (0.14-0.80)**	0.013*
(n = 257)		AA	5 (15.6)	38 (16.9)	0.57 (0.20-1.64)	0.294
		GA+AA	13 (40.6)	143 (63.6)	**0.39 (0.18-0.84)**	0.015*
						
	*AKT1*	CC+G	14 (43.8)	53 (23.6)	Reference	
	rs2494750	CC+A	13 (40.6)	115 (51.1)	**0.43 (0.19-0.97)**	0.043*
	**+**	CG+G	2 (6.3)	25 (11.1)	0.30 (0.06-1.44)	0.132
	*AR*	CG+A	3 (9.4)	31 (13.8)	0.37 (0.10-1.38)	0.137
	rs17302090	GG+A	0 (0.0)	1 (0.4)	–	–
						
	*AKT1*	CC+GG	16 (50.0)	57 (25.3)	Reference	
	rs2494750	CC+GA	7 (21.9)	81 (36.0)	**0.31 (0.12-0.80)**	0.015*
	**+**	CC+AA	4 (12.5)	30 (13.3)	0.48 (0.15-1.55)	0.217
	*AMACR*	CG+GG	3 (9.4)	25 (11.1)	0.43 (0.11-1.60)	0.207
	rs3195676	CG+GA	1 (3.1)	23 (10.2)	0.16 (0.02-1.24)	0.078
		CG+AA	1 (3.1)	8 (3.6)	0.45 (0.05-3.83)	0.461
		GG+GA	0 (0.0)	1 (0.4)	–	–
						
Bilaterality	*AKT1*	CC+G	43 (31.4)	24 (19.8)	Reference	
(n = 258)	rs2494750	CC+A	63 (46.0)	66 (54.5)	**0.53 (0.29-0.98)**	0.042*
	**+**	CG+G	13 (9.5)	14 (11.6)	0.52 (0.21-1.28)	0.155
	*AR*	CG+A	17 (12.4)	17 (14.0)	0.56 (0.24-1.29)	0.172
	rs17302090	GG+G	0 (0.0)	0 (0.0)	–	–
		GG+A	1 (0.7)	0 (0.0)	–	
						
	*PI3K*	GG+G	38 (27.7)	21 (17.4)	Reference	
	rs2699887	GG+A	47 (34.3)	52 (43.0)	**0.50 (0.26-0.97)**	0.040*
	**+**	GA+G	18 (13.1)	16 (13.2)	0.62 (0.26-1.47)	0.278
	*AR*	GA+A	26 (19.0)	25 (20.7)	0.58 (0.27-1.24)	0.156
	rs17302090	AA+G	0 (0.0)	1 (0.8)	–	–
		AA+A	8 (5.8)	6 (5.0)	0.74 (0.23-2.41)	0.614
Extracapsular	*PTEN*	CC+GG	6 (6.2)	22 (13.8)	Reference	
Extension	rs2735343	CC+GA	6 (6.2)	11 (6.9)	2.00 (0.52-7.66)	0.312
(n = 257)	**+**	CC+AA	3 (3.1)	3 (1.9)	3.67 (0.58-23.03)	0.166
	*AMACR*	CG+GG	16 (16.5)	19 (11.9)	**3.09 (1.01-9.48)**	0.049*
	rs3591676	CG+GA	20 (20.6)	30 (18.8)	2.44 (0.84-7.09)	0.100
		CG+AA	11 (11.3)	13 (8.1)	3.10 (0.93-10.39)	0.066
		GG+GG	15 (15.5)	23 (14.4)	2.39 (0.79-7.28)	0.125
		GG+GA	17 (17.5)	29 (18.1)	2.15 (0.73-6.35)	0.166
		GG+AA	3 (3.1)	10 (6.3)	1.10 (0.23-5.31)	0.906
						
	*PTEN*	CC+G	5 (5.2)	21 (13.1)	Reference	
	rs2735343	CC+A	10 (10.3)	15 (9.4)	2.80 (0.79-9.89)	0.110
	**+**	CG+G	15 (15.5)	18 (11.3)	**3.50 (1.06-11.53)**	0.039*
	*AR*	CG+A	32 (33.0)	44 (27.5)	**3.06 (1.04-8.96)**	0.042*
	rs17302090	GG+G	14 (14.4)	21 (13.1)	2.80 (0.86-9.18)	0.089
		GG+A	21 (21.6)	41 (25.6)	2.15 (0.71-6.52)	0.175

OR (CI95%): Odds Ratio value, with a confidence interval of 95%.

* Statistically significant value, *p*< 0.05.

When the genotypes of different SNPs were compared, several statistically significant results were obtained. The following associations indicated protection for seminal vesicle invasion: *AKT1* (CC) *+ AR* (A) and *AKT1* (CC) + *AMACR* (GA). The associations that indicated protection for tumor bilaterality were: *AKT1* (CC) + *AR* (A) and *PI3K* (GG) + *AR* (A) (Table 5).

Finally, the following combinations of genotypes indicated a greater risk of extracapsular extension: *PTEN* (CG) + *AMACR* (GG), *PTEN* (CG) + *AR* (G), and *PTEN* (CG) + *AR* (A) (Table 5).

## Discussion

PCa is a heterogeneous pathology that currently has a lifetime possibility of detection of 19%, while the risk of death is just 8%. This discrepancy between incidence and mortality is attributed not just to treatment offered, but also to the increase in initial tumor detection and slow growth (Siegel *et al.*, 2017). Patients with slow-growing tumors could be exposed to unnecessary risks as a consequence of therapy. As such, currently, the greatest challenge with PCa is the identification of markers that can be used to classify tumors according to their potential for aggressive behavior and the recognition of patients that need specific therapy (Vanacore *et al.*, 2017).

The rate of development of PCa varies substantially according to age (Rebbeck, 2017), ancestry (Bell *et al.*, 2015), and family history (Siegel *et al.*, 2017). In this study, a family history of cancer was a risk factor (*p* = 0.001). Patients with PCa in their family also presented greater susceptibility to developing prostatic neoplasia (*p* = 0.031). These findings demonstrate the high contribution of heredity in the development of this carcinoma. Cremers *et al.* (2016) demonstrated that, while there are inheritable sites that determine susceptibility to prostate cancer, there is not only one gene that alone determines susceptibility; the pathology of the disease involves a large number of genes, meaning that this illness is treated as being multifactorial.

Education is an indicator of socioeconomic status, and is inversely associated with the incidence of cancer in various organs; in general, the greater the level of schooling, the lower the risk of aggressive cancer (Mouw *et al.*, 2008). In the studied sample, the individuals without formal schooling presented twice the risk of PCa when compared to their respective controls. These data are consistent with Wolf *et al.* (2006), who stated that a higher degree of schooling offers greater comprehension of prostate health and, consequently, leads to individuals seeking out medical care. In this case, low patient schooling could lead to tumor diagnosis in more advanced stages and result in a higher mortality index.

The average patient age in this study was 65.38 ± 7.15 years, which corroborates the literature data that show that PCa is a disease of old age; the majority of cases of sporadic PCa are observed in men over the age of 65. This fact has been related to the accumulation of DNA damage (O'Connor, 2015).

PSA is a glycoprotein produced by prostate epithelial cells that does not constitute a specific cancer marker, given that it can be changed in other diseases of the prostate (Hamdy *et al.*, 2016; Hendriks *et al.*, 2017); furthermore, no exact value exists that can determine the presence of a malignant disease. The PSA values at the time of diagnosis determined in this study ranged from 1.5 to 933.9 ng/mL, confirming that the specificity of this marker for PCa is low. Given this characteristic, the U.S. Preventive Services Task Force, as well as various other health entities around the world, recommend not tracking PCa using PSA, given that despite appearing in many PCa cases, its correlation with mortality is minimal (Jemal *et al.*, 2015).

Considering the important role that SNPs play as biomarkers in various types of diseases, genes and SNPs that are candidates for PCa susceptibility and prognosis were chosen (Wang *et al.*, 2016). These include PTEN/PI3K/AKT signaling pathway components, which are important regulators for growth, metabolism, cell cycle, DNA repair, and apoptosis inhibition (Zhu *et al.*, 2016).

The rs2735343 gene *PTEN*, studied herein is located in an intronic region. According to Jang *et al.* (2013), it can influence splicing, protein expression, and, consequently, the regulation of the cell cycle and the risk of cancer. The aforementioned authors showed that carriers of the homozygous GG genotype, the same SNP evaluated in this study, presented a greater risk for esophageal squamous cell carcinoma (ESCC). This SNP was also considered by Ma *et al.* (2012) as a factor of susceptibility for ESCC in patients that presented CG and GG genotypes. Song *et al.* (2017) performed a meta-analysis with different types of cancer, including PCa and the associated risk of cancer for carriers of the G allele in an Asian population. In this study, we did not find any association between the variants of this gene and susceptibility to PCa. Nonetheless, in all genotypic combinations in which we detected a greater risk of extracapsular extension, the G allele of the (rs2735343) *PTEN* gene was present (Table 4), which seems to confirm the above-cited hypotheses.

The heterozygous genotype (CG) in these cases can create a non-functional protein, given that, according to Jang *et al.* (2013), this tumor suppressor gene presents very unusual characteristics. As opposed to the majority of tumor suppressor genes, the loss of just a single allele results in an abnormal phenotype, i.e., it is considered a haploinsufficient suppressor and, as a result, does not follow the classical genetic model of biallelic inactivation. The loss of activity of this protein seems to be common in different types of cancer (Inoue and Fry, 2017) and explains our findings.

Several studies have demonstrated that tumors with extracapsular extension had a higher risk of progression than those confined to the prostate and this can be considered an indicator of poor prognosis (Troyer *et al.*, 2015; Epstein *et al.*, 2016). Troyer *et al.* (2015) reported that a PTEN deletion was significantly associated with higher extracapsular extension. Our results, added to these, suggest that alterations in this gene have the ability to predict aggressive disease.

The gene *AKT1* belongs to the *AKT* family and the protein that it codes plays an important role in cellular survival via apoptosis control; it is related to the development of various diseases, including cancer (Painter *et al.*, 2016). The relationship between PCa and rs2494750SNP is controversial. Karyadi *et al.* (2015) and Fitzgerald *et al.* (2018) associated this SNP with death specifically for PCa patients carrying allele G. Nonetheless, this SNP, in the present study, protected the carriers of the CG genotypes against PCa. The heterozygous genotype, when combined with the A allele of the (rs17302090) *AR* gene, also protects against PCa. Other research has failed to associate the (rs2494750) *AKT1* gene with squamous cell carcinoma of the esophagus (Zhu *et al.*, 2016) or with cancer of the endometrium (Fallah *et al.*, 2015). Given these conflicting results, it would be interesting to investigate this question further with larger samples of PCa patients from other populations.

Studies, both *in vitro* and *in vivo*, have demonstrated that PI3K/AKT/mTOR signaling interacts with the *AR* gene in an antagonistic manner; when the pathway is inhibited, it increases expression of AR, and as such could serve as a therapeutic target for patients with androgen-dependent prostate cancer (Kaarbø *et al.*, 2010). According to these authors, it appears that the *AR* gene signaling pathway, depending on downstream genes, could be involved in the malignant transformation of PCa.

Some of the SNPs studied in the *AR* gene have already demonstrated that the coded protein changes binding with cofactors. This change is associated with androgen insensitivity syndrome, which can affect the activity and level of circulating androgens (Naeyer *et al.*, 2014). In our study, the rs17302090 SNP was not associated with PCa. Sun *et al.* (2010) evaluated the rs1204038, which presents strong linkage disequilibrium with rs17302090, evaluated in this study, and did not identify any association between risk of aggressive PCa and androgen deprivation therapy. On the other hand, Lindström *et al.* (2007) demonstrated that carriers of the rare allele A (rs17302090) who received primary hormonal therapy presented twice the risk of death from PCa compared to individuals that had the prevalent allele. The authors further suggested that this polymorphism would act as a modifier in the hormonal treatment of the disease leading to death by PCa.

Nonetheless, allele A of the *AR* gene, when combined with the gene *AKT1* (CC), conferred protection against seminal vesicle invasion and tumor bilaterality; when combined with the gene *PI3K* (GG), it also conferred protection against tumor bilaterality. The finding of seminal vesicle invasion at the time of radical prostatectomy is an adverse pathologic finding that confers a decrease in long-term freedom from biochemical recurrence, exceeded in magnitude only by the finding of lymph node metastases (Potter *et al.*, 2000).

The last gene evaluated in this study codes the enzyme AMACR, which is overexpressed in prostate tumor tissue. This overexpression seems to be involved in carcinoma initiation, as this type of alteration has been described in prostatic intraepithelial neoplasia (Jarvis *et al.*, 2015; Jemal *et al.*, 2015). The enzyme AMACR participates in the metabolism of fatty acids. The main dietary source of branched-chain fatty acids in the human diet includes red meat, which is considered a PCa risk factor. The consumption of these fatty acids is associated with the overexpression of *AMACR* in cancerous prostate cells, as it consistently increases oxidation in these cells, resulting in the production of reactive oxygen species that can damage DNA (Wright *et al.*, 2011).

The rs3195676 SNP, located on exon 1 in a coding region, results in the substitution of valine for methionine. The use of bioinformatics tools to predict the influence of this exchange of amino acids on protein integrity has demonstrated that this substitution is not tolerated in carriers of recessive alleles of this SNP, suggesting that this results in protein damage and lower expression (Levin *et al.*, 2007).

Lee *et al.* (2013) studied this polymorphism and demonstrated that male carriers of allele G with a Gleason score ≥ 7 and ≥ pT2c present a greater risk of developing PCa. An *in vitro* experiment also demonstrated that a decrease in the level of AMACR protein results in a decrease in the proliferation of prostate adenocarcinoma cells (Zha *et al.*, 2003). This explains the results obtained herein, given that whenever the A allele of the SNP was present, it provided protection against more aggressive PCa - the AMACR protein was damaged and likely less expressed, improving the prognosis for the patient carriers of the rare allelei n relation to seminal vesicle invasion. Genotypes of the AMACR gene (GA/GA+AA) alone or in combination with the AKT1 gene (CC) significantly protected the occurrence of seminal vesicle invasion.

**Figure 1:**
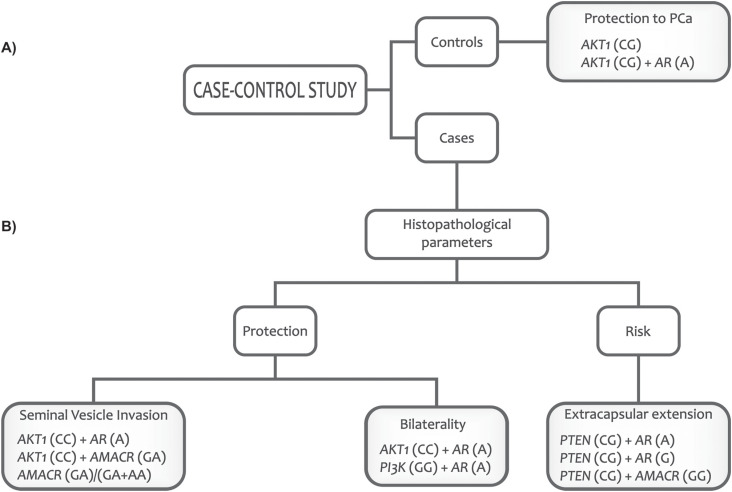
An individual's genetic profile can: (A) Identify men with genotypes that confer protection for prostate cancer. (B) Discriminate men with a better or worse prognosis for prostate cancer.

The *AKT1* and *AR* genes, alone or combined, showed protection for PCa, while *AKT1, PI3K, AR*, and *AMACR* genes protected against PCa aggressiveness. Only the *PTEN* gene associated with *AMACR* or *AR* indicated a risk for extracapsular extension. These results, showing the influence of the SNPs assessed on the clinical and pathological outcomes of PCa, are summarized in Figure 1.

The identification and implementation of novel molecular markers to improve the prediction of tumor behavior in patients with PCa will reduce the variability among observers regarding the distinction of many histopathological parameters. This is important for treatment decisions that depend heavily on the opinions of an individual pathologist (Hoogland *et al.*, 2014). The results of our study indicate SNPs as potential molecular markers to be used in prognostic panels for PCa, however, these findings still need additional confirmation before use in clinical practice.

## Supplementary material

The following online material is available for this article:

Table S1 Univariate and multivariate regression analysis of association of genotypes of *PTEN, AKT, PI3K, AR*, and *AMACR* genes with prostate cancer risk.

Table S2 Association between genotypes of *PTEN, AKT, PI3K, AR*, and *AMACR* genes and clinicopathological features in prostate cancer patients.
