# Supplementary material for: Association of polymorphisms of PTEN, AKT1, PI3K, AR, and AMACR genes in patients with prostate cancer
Source: Genet Mol Biol. 2020 Jun 1;43(3):e20180329. doi: 10.1590/1678-4685-GMB-2018-0329 (PMC7271063; doi:10.1590/1678-4685-GMB-2018-0329)
Supplement: Supplementary file 1 [file 1415-4757-GMB-43-3-e20180329-s1.pdf]

Supplementary Material to "Association of polymorphisms of *PTEN*, *AKT1*, *PI3K*, *AR*, and *AMACR* genes in patients with prostate cancer"

Table S1 – Univariate and multivariate regression analysis of association of genotypes of *PTEN*, *AKT*, *PI3K*, *AR*, and *AMACR* genes with prostate cancer risk.

Gene SNP_ID	Genotypes	Cases N (%)	Controls N (%)	Not adjusted		Adjusted	
				OR (CI95%)	*p*-value	OR (CI95%)	*p*-value
PTEN	CC+GG	35 (12.6)	23 (8.3)	Reference		Reference	
rs2735343	CC+GA	16 (5.8)	10 (3.6)	1.05 (0.41-2.72)	0.918	1.20 (0.21-7.02)	0.838
+	CC+AA	4 (1.4)	3 (1.1)	0.88 (0.18-4.28)	0.870	-	-
PI3K	GC+GG	72 (26)	96 (34.7)	**0.49 (0.27-0.91)**	**0.023***	0.46 (0.16-1.31)	0.148
rs2699887	GC+GA	35 (12.6)	39 (14.1)	0.59 (0.29-1.18)	0.137	0.46 (0.14-1.47)	0.189
	GC+AA	8 (2.9)	8 (2.9)	0.66 (0.22-2.00)	0.459	0.57 (0.10-3.33)	0.531
	GG+GG	65 (23.5)	58 (20.9)	0.74 (0.39-1.39)	0.344	0.63 (0.22-1.81)	0.389
	GG+GA	39 (14.1)	38 (13.7)	0.67 (0.34-1.34)	0.263	0.56 (0.18-1.77)	0.324
	GG+AA	3 (1.1)	2 (0.7)	0.99 (0.15-6.36)	0.988	-	-
PTEN	CC+CC	45 (16.2)	30 (10.8)	Reference		Reference	
rs2735343	CC+CG	10 (3.6)	6 (2.2)	1.11 (0.36-3.38)	0.853	1.32 (0.24-7.35)	0.749
+	CG+CC	82 (29.6)	99 (35.7)	**0.55 (0.32-0.95)**	**0.033***	0.55 (0.20-1.49)	0.242
AKT1	CG+CG	32 (11.6)	44 (15.9)	**0.48 (0.25-0.93)**	**0.029***	0.30 (0.09-0.98)	0.056
rs2494750	CG+GG	1 (0.4)	0 (0.0)	-	-	-	-
	GG+CC	83 (30)	74 (26.7)	0.75 (0.43-1.31)	0.307	0.63 (0.24-1.72)	0.634
	GG+CG	24 (8.7)	23 (8.3)	0.70 (0.33-1.45)	0.333	0.59 (0.15-2.24)	0.585
	GG+GG	0 (0.0)	1 (0.4)	-	-	-	-

**Gene**<br>**SNP_ID**	**Genotypes**	**Cases**<br>**N (%)**	**Controls**<br>**N (%)**	**Not adjusted**		**Adjusted**	
				**OR (CI95%)**	***p*-value**	**OR (CI95%)**	***p*-value**
PTEN	CC+GG	19 (6.9)	11 (4.0)	Reference		Reference	
rs2735343	CC+GA	30 (10.8)	16 (5.8)	0.92 (0.35-2.40)	0.867	1.56 (0.31-7.84)	0.591
+	CC+AA	6 (2.2)	9 (3.2)	0.36 (0.11-1.18)	0.091	1.24 (0.07-21.81)	0.882
AMACR	CG+GG	55 (19.9)	70 (25.3)	**0.46 (0.21-0.97)**	**0.041***	0.55 (0.16-1.89)	0.346
rs3195676	CG+GA	36 (13)	42 (15.2)	**0.42 (0.21-0.85)**	**0.015***	0.48 (0.15-1.50)	0.204
	CG+AA	24 (8.7)	31 (11.2)	**0.41 (0.18-0.93)**	**0.032***	0.53 (0.14-1.99)	0.350
	GG+GG	51 (18.4)	37 (13.4)	0.57 (0.27-1.22)	0.149	0.78 (0.23-2.64)	0.692
	GG+GA	41 (14.8)	38 (13.7)	0.73 (0.35-1.54)	0.415	1.08 (0.32-3.59)	0.906
	GG+AA	15 (5.4)	23 (8.3)	**0.35 (0.14-0.85)**	**0.020***	0.20 (0.05-0.87)	0.310
PTEN	CC+G	28 (10.1)	15 (5.4)	Reference		Reference	
rs2735343	CC+A	27 (9.7)	21 (7.6)	0.69 (0.29-1.61)	0.389	1.65 (0.33-8.36)	0.545
+	CG+G	34 (12.3)	39 (14.1)	0.47 (0.21-1.02)	0.055	0.55 (0.15-2.07)	0.378
AR	CG+A	81 (29.2)	104 (37.5)	**0.42 (0.21-0.83)**	**0.013***	0.54 (0.17-1.73)	0.298
rs1730209	GG+G	37 (13.4)	34 (12.3)	0.58 (0.27-1.27)	0.176	0.98 (0.26-3.78)	0.977
	GG+A	70 (25.3)	64 (23.1)	0.57 (0.29-1.19)	0.142	(0.19-2.04)	0.433
AKT1	CC+GG	129 (46.6)	135 (48.7)	Reference		Reference	
rs2494750	CC+GA	71 (25.6)	58 (20.9)	1.28 (0.84-1.95)	0.251	1.19 (0.60-2.35)	0.625
+	CC+AA	10 (3.6)	10 (3.6)	1.05 (0.42-2.60)	0.922	1.28 (0.31-5.27)	0.729
PI3K	CG+GG	42 (15.2)	41 (14.8)	1.07 (0.65-1.76)	0.782	0.99 (0.45-2.75)	0.971
rs2699887	CG+GA	19 (6.9)	29 (10.5)	0.69 (0.37-1.28)	0.238	0.47 (0.16-1.35)	0.158
	CG+AA	5 (1.8)	3 (1.1)	1.74 (0.41-7.45)	0.453	-	-
	GG+GG	1 (0.4)	1 (0.4)	1.05 (0.06-16.91)	0.974	-	-

Gene SNP_ID	Genotypes	Cases N (%)	Controls N (%)	Not adjusted		Adjusted	
				OR (CI95%)	*p*-value	OR (CI95%)	*p*-value
AKT1	CC+GG	78 (28.2)	71 (25.6)	Reference		Reference	
rs2494750	CC+GA	96 (34.7)	79 (28.5)	1.11 (0.71-1.71)	0.652	1.14 (0.58-2.28)	0.699
+	CC+AA	36 (13)	53 (19.1)	0.62 (0.36-1.05)	0.076	0.59 (0.25-1.38)	0.223
AMACR	CG+GG	29 (10.5)	25 (9)	1.06 (0.57-1.97)	0.864	0.99 (0.39-2.49)	0.975
rs3195676	CG+GA	28 (10.1)	38 (13.7)	0.67 (0.37-1.20)	0.180	0.65 (0.24-1.77)	0.395
	CG+AA	9 (3.2)	10 (3.6)	0.82 (0.32-2.13)	0.683	0.32 (0.07-1.46)	0.142
	GG+GA	1 (0.4)	1 (0.4)	0.91 (0.06-14.83)	0.947	-	-
AMACR	GG+G	99 (35.7)	88 (31.8)	Reference		Reference	
rs3195676	GG+A	8 (2.9)	8 (2.9)	0.90 (0.32-2.47)	0.821	0.68 (0.09-5.00)	0.708
+	GA+A	125 (45.1)	118 (42.6)	0.94 (0.64-1.38)	0.757	1.00 (0.49-2.01)	0.990
AR	AA+G	0 (0.0)	0 (0.0)	-	-	-	-
rs17302090	AA+A	45 (16.2)	63 (22.7)	0.63 (0.39-1.02)	0.063	0.52 (0.21-1.25)	0.141
PI3K	GG+G	62 (22.4)	57 (20.6)	Reference		Reference	
rs2699887	GG+A	110 (39.7)	120 (43.3)	0.84 (0.54-1.31)	0.449	0.97 (0.44-2.18)	0.949
+	GA+G	36 (13)	27 (9.7)	1.23 (0.66-2.27)	0.516	1.73 (0.53-5.67)	0.364
AR	GA+A	54 (19.5)	60 (21.7)	0.83 (0.49-1.38)	0.470	0.68 (0.27-1.71)	0.418
rs1730209	AA+G	1 (0.4)	4 (1.4)	0.23 (0.02-2.12)	0.194	-	-
	AA+A	14 (5.1)	9 (3.2)	1.43 (0.57-3.56)	0.442	2.79 (0.37-21.08)	0.319
PI3K	GG+GG	66 (23.8)	59 (21.3)	Reference		Reference	
rs2699887	GG+GA	81 (29.2)	77 (27.8)	0.94 (0.59-1.50)	0.798	1.27 (0.55-2.91)	0.578

**Gene SNP_ID**	**Genotypes**	**Cases N (%)**	**Controls N (%)**	**Not adjusted**		**Adjusted**	
				**OR (CI95%)**	***p*-value**	**OR (CI95%)**	***p*-value**
+	GG+AA	25 (9.0)	41 (14.8)	0.55 (0.30-1.00)	0.051	0.54 (0.19-1.54)	0.246
AMACR	GA+GG	40 (14.4)	33 (11.9)	1.08 (0.61-1.93)	0.786	1.54 (0.53-4.44)	0.424
rs3195676	GA+GA	33 (11.9)	39 (14.1)	0.76 (0.42-1.35)	0.347	0.52 (0.13-2.06)	0.597
	GA+AA	17 (6.1)	15 (5.4)	1.01 (0.47-2.21)	0.947	0.52 (0.13-2.06)	0.353
	AA+GG	1 (0.4)	4 (1.4)	0.22 (0.02-2.06)	0.186	-	-
	AA+GA	11 (4.0)	2 (0.7)	**4.92 (1.04-23.10)**	**0.044***	3.58 (0.27-47.91)	0.335
	AA+AA	3 (1.1)	7 (2.5)	0.38 (0.10-1.55)	0.178	2.03 (0.13-32.91)	0.619

OR (CI95%): *Odds Ratio* value, with a confidence interval of 95%.

Adjusted for family history of cancer, family history of prostate cancer, and schooling.

*Statistically significant value, $p < 0.05$.
