# Supplementary material for: Association of polymorphisms of PTEN, AKT1, PI3K, AR, and AMACR genes in patients with prostate cancer
Source: Genet Mol Biol. 2020 Jun 1;43(3):e20180329. doi: 10.1590/1678-4685-GMB-2018-0329 (PMC7271063; doi:10.1590/1678-4685-GMB-2018-0329)
Supplement: Supplementary file 2 [file 1415-4757-GMB-43-3-e20180329-s2.pdf]

Supplementary Material to "Association of polymorphisms of *PTEN*, *AKT1*, *PI3K*, *AR*, and *AMACR* genes in patients with prostate cancer"

Table S2 – Association between genotypes of *PTEN*, *AKT*, *PI3K*, *AR*, and *AMACR* genes and clinicopathological features in prostate cancer patients

		Bilaterality (N=258)			Extracapsular extension (N=257)		
**Genes SNP_ID**	**Genotypes**	**P N (%)**	**A N (%)**	**OR (CI95%)**	**P N (%)**	**A N (%)**	**OR (CI95%)**
PTEN	CC	27 (19.7)	24 (19.8)	Reference	15(15.5)	36 (22.5)	Reference
rs2735343	CG	57 (41.6)	53 (43.8)	0.96 (0.49-1.86)	47 (48.5)	62 (38.8)	1.82 (0.89-3.71)
	GG	53 (38.7)	44 (36.4)	1.07 (0.54-2.11)	35 (36.1)	62 (38.8)	1.36 (0.65-2.82)
	CG+GG	110 (80.3)	97 (80.2)	1.01 (0.55-1.86)	82 (84.5)	124 (77.5)	1.59 (0.82-3.08)
AKT1	CC	106 (77.4)	90 (74.4)	Reference	77 (79.4)	118 (73.8)	Reference
rs2494750	CG	30 (21.9)	31 (25.6)	0.82 (0.46-1.46)	20 (20.6)	41 (25.6)	0.75 (0.41-1.37)
	GG	1 (0.7)	-	-	0 (0.0)	1 (0.6)	-
	CG+GG	31 (22.6)	31 (25.6)	0.85 (0.48-1.50)	20 (20.6)	42 (26.3)	0.73 (0.40-1.34)
**PI3K**	GG	85 (62.0)	73 (60.3)	Reference	61 (62.9)	96 (60.0)	Reference
rs2699887	GA	44 (32.1)	41 (33.9)	0.92 (0.54-1.56)	31 (32.0)	54 (33.8)	0.90 (0.52-1.56)
	AA	8 (5.8)	7 (5.8)	0.98 (0.34-2.84)	5 (5.2)	10 (6.3)	0.79 (0.26-2.41)
	GA+AA	52 (38.0)	48 (39.7)	0.93 (0.56-1.54)	36 (37.1)	64 (40.0)	0.89 (0.53-1.49)
AMACR	GG	60 (43.8)	41 (33.9)	Reference	37 (38.1)	64 (40.0)	Reference
rs3591676	GA	58 (42.3)	56 (46.3)	0.71 (0.41-1.22)	43 (44.3)	70 (43.8)	1.06 (0.61-1.85)
	AA	19 (13.9)	24 (19.8)	0.54 (0.26-1.11)	17 (17.5)	26 (16.3)	1.13 (0.54- 2.34)
	GA+AA	77 (56.2)	80 (66.1)	0.66 (0.40-1.09)	60 (61.9)	96 (60.0)	1.11 (0.65-1.88)
**AR**	G	56 (40.9)	38 (31.4)	Reference	34 (35.1)	60 (37.5)	Reference
rs17302090	A	81 (59.1)	83 (68.6)	0.66 (0.40-1.11)	63 (64.9)	100 (62.5)	1.11 (0.66-1.88)
PTEN	CC	8 (25.0)	43 (19.1)	Reference	7 (16.7)	44 (20.5)	Reference
rs2735343	CG	12 (37.5)	97 (43.1)	0.67 (0.25-1.74)	21 (50.0)	88 (40.9)	1.50 (0.59-3.80)
	GG	12 (37.5)	85 (37.8)	0.76 (0.29-2.00)	14 (33.3)	83 (38.6)	1.06 (0.40-2.82)
	CG+GG	24 (75.0)	182 (80.9)	0.71 (0.30-1.69)	35 (83.3)	171 (79.5)	1.29 (0.54-3.09)
AKT1	CC	27 (84.4)	168 (74.7)	Reference	30 (71.4)	165 (76.7)	Reference
rs2494750	CG	5 (15.6)	56 (24.9)	0.56 (0.20-1.48)	11 (26.2)	50 (23.3)	1.21 (0.57-2.59)
	GG	0 (0.0)	1 (0.4)	-	1 (2.4)	0 (0.0)	-
	CG+GG	5 (15.6)	57 (25.3)	0.55 (0.20-1.51)	12 (28.6)	50 (23.3)	1.32 (0.63-2.77)
**PI3K**	GG	19 (59.4)	138 (61.3)	Reference	30 (71.4)	127 (59.1)	Reference
rs2699887	GA	12 (37.5)	73 (32.4)	1.19 (0.55-2.60)	11 (26.2)	74 (34.4)	0.63 (0.30-1.33)
	AA	1 (3.1)	14 (6.2)	0.52 (0.07-4.17)	1 (2.4)	14 (6.5)	0.30 (0.04-2.39)
	GA+AA	13 (40.6)	87 (38.7)	1.09 (0.51-2.31)	12 (28.6)	88 (40.9)	0.58 (0.28-1.20)
AMACR	GG	19 (59.4)	82 (36.4)	Reference	20 (47.6)	81 (37.7)	Reference
rs3591676	GA	8 (25.0)	105 (46.7)	**0.32 (0.13-0.78)***	14 (33.3)	99 (46.0)	0.57 (0.27-1.21)
	AA	5 (15.6)	38 (16.9)	0.56 (0.19-1.63)	8 (19.0)	35 (16.3)	0.93 (0.37-2.30)
	GA+AA	13 (40.6)	143 (63.6)	**0.39 (0.18-0.83)***	22 (52.4)	134 (62.3)	0.67 (0.34-1.29)
**AR**	G	16 (50.0)	68 (34.7)	Reference	18 (42.9)	76 (35.3)	Reference
rs17302090	A	16 (50.0)	147 (65.3)	0.53 (0.25-1.12)	24 (57.1)	139 (64.7)	0.73 (0.37-1.43)

		Bilaterality (N=258)			Extracapsular extension (N=257)		
**Genes SNP_ID**	**Genotypes**	**P N (%)**	**A N (%)**	**OR (CI95%)**	**P N (%)**	**A N (%)**	**OR (CI95%)**
PTEN	CC	35 (22.7)	16 (15.8)	Reference	31 (19.7)	24 (20.9)	Reference
rs2735343	CG	58 (37.7)	50 (49.5)	1.89 (0.94-3.81)	63 (40.1)	50 (43.5)	1.03 (0.54-1.96)
	GG	61 (39.6)	35 (34.7)	1.26 (0.61-2.59)	63 (40.1)	41 (35.7)	0.84 (0.43-1.63)
	CG+GG	119 (77.3)	85 (84.2)	1.56 (0.81-3.00)	126 (80.3)	91 (79.1)	0.93 (0.51-1.70)
AKT1	CC	114 (74.0)	80 (79.2)	Reference	122 (77.7)	85 (73.9)	Reference
rs2494750	CG	40 (26.0)	21 (20.8)	0.75 (0.41-1.36)	34 (21.7)	30 (26.1)	1.27 (0.72-2.23)
	GG	0 (0.0)	0 (0.0)	-	1 (0.6)	0 (0.0)	0.99 (-)
	CG+GG	40 (26.0)	21 (20.8)	0.75 (0.41-1.36)	35 (22.3)	30 (26.1)	1.23 (0.70-2.16)
PI3K	GG	94 (61.0)	62 (61.4)	Reference	101 (64.3)	68 (59.1)	Reference
rs2699887	GA	51 (33.1)	34 (33.7)	1.01 (0.59-1.73)	51 (32.5)	37 (32.2)	1.08 (0.64-1.82)
	AA	9 (5.8)	5 (5.0)	0.84 (0.27-2.63)	5 (3.2)	10 (8.7)	2.97 (0.97-9.07)
	GA+AA	60 (39.0)	39 (38.6)	0.96 (0.59-1.65)	56 (35.7)	47 (40.9)	1.25 (0.76-2.05)
AMACR	GG	61 (39.6)	40 (39.6)	Reference	59 (37.6)	47 (40.9)	Reference
rs3591676	GA	66 (42.9)	45 (44.6)	1.01 (0.59-1.65)	69 (43.9)	54 (47.0)	0.95 (0.58-1.66)
	AA	27 (17.5)	16 (15.8)	0.84 (0.27-2.63)	29 (18.5)	14 (12.2)	0.61 (0.29-1.28)
AR	G	58 (37.7)	36 (35.6)	Reference	54 (38.3)	44 (34.4)	Reference
rs17302090	A	96 (62.3)	65 (64.4)	1.09 (0.65-1.84)	103 (65.6)	71 (61.7)	0.85 (0.51-1.40)
PTEN	CC	35 (22.7)	16 (15.8)	Reference	31 (19.7)	24 (20.9)	Reference

				Gleason (N=275)
**Genes** SNP_ID	**Genotypes**	**3 to 7 (3+4) N (%)**	**7 (4+3) to 10 N (%)**	**Genes** SNP_ID
PTEN	CC	47 (20.5)	8 (17.4)	***PTEN***
rs2735343	CG	95 (41.5)	19 (41.3)	rs2735343
	GG	87 (38.0)	19 (41.3)	
	CG+GG	182 (79.5)	38 (82.6)	
AKT1	CC	171 (74.7)	37 (80.4)	***AKT1***
rs2494750	CG	57 (24.9)	9 (19.6)	rs2494750
	GG	1 (0.4)	0 (0.0)	
	CG+GG	58 (25.3)	9 (19.6)	
PI3K	GG	143 (62.4)	28 (60.9)	***PI3K***
rs2699887	GA	74 (32.3)	15 (32.6)	rs2699887
	AA	12 (5.2)	3 (6.5)	
	GA+AA	86 (37.6)	18 (39.1)	
AMACR	GG	86 (37.6)	20 (43.5)	***AMACR***
rs3591676	GA	104 (45.4)	20 (43.5)	rs3591676
	AA	39 (17.0)	6 (13.0)	
	GA+AA	143 (62.4)	26 (56.5)	
AR	G	79 (34.5)	19 (41.3)	***AR***
rs17302090	A	150 (65.5)	27 (58.7)	rs17302090

OR (CI95%): Odds Ratio value, with a confidence interval of 95%;
*Statistically significant value, $p < 0.05$.
